# Parasite clearance following treatment with sulphadoxine-pyrimethamine for intermittent preventive treatment in Burkina-Faso and Mali: 42-day in vivo follow-up study

**DOI:** 10.1186/1475-2875-13-41

**Published:** 2014-01-31

**Authors:** Sheick O Coulibaly, Kassoum Kayentao, Steve Taylor, Etienne A Guirou, Carole Khairallah, Nouhoun Guindo, Moussa Djimde, Richard Bationo, Alamissa Soulama, Edgar Dabira, Binta Barry, Moussa Niangaly, Hammadoun Diakite, Sidiki Konate, Mohamed Keita, Boubacar Traore, Steve R Meshnick, Pascal Magnussen, Ogobara K Doumbo, Feiko O ter Kuile

**Affiliations:** 1 Faculty of Health, University of Ouagadougou, Ouagadougou, Burkina Faso; 2 Department of Epidemiology of Parasitic Diseases, Malaria Research and Training Center, Faculty of Medicine and Odonto-stomatology of Bamako, University of Sciences, Technics and Technologies, BP: 1805, Bamako, Mali; 3 Malaria Epidemiology Unit, Department of Clinical Sciences, Liverpool School of Tropical Medicine, Liverpool, UK; 4 Division of Infectious Diseases & International Health Duke University Medical Center, Durham, NC, USA; 5 Department of Epidemiology, Gillings School of Global Public Health, University of North Carolina, Chapel Hill, NC, USA; 6 Centre for Medical Parasitology, Faculty of Health and Medical Sciences, University of Copenhagen, Copenhagen, Denmark

**Keywords:** Malaria, Pregnancy, Intermittent, Sulphadoxine-pyrimethamine, Resistance, Mali, Burkina-Faso

## Abstract

**Background:**

Intermittent Preventive Treatment in pregnancy (IPTp) with sulphadoxine-pyrimethamine (SP) is widely used for the control of malaria in pregnancy in Africa. The emergence of resistance to SP is a concern requiring monitoring the effectiveness of SP for IPTp.

**Methods:**

This was an *in-vivo* efficacy study to determine the parasitological treatment response and the duration of post-treatment prophylaxis among asymptomatic pregnant women receiving SP as part of IPTp in Mali and Burkina-Faso. The primary outcome was the PCR-unadjusted % of patients with parasites recurrence by day 42 defined as a positive diagnostic test by malaria smear at any visit between days 4 and 42. Treatment failure was based on the standard World Health Organization criteria. The therapeutic response was estimated using the Kaplan-Meier curve.

**Results:**

A total of 580 women were enrolled in Mali (N=268) and Burkina-Faso (N=312) and followed weekly for 42 days. Among these, 94.3% completed the follow-up. The PCR-unadjusted cumulative risk of recurrence by day 42 was 4.9% overall, and 3.2% and 6.5% in Mali and Burkina Faso respectively (Hazard Ratio [HR] =2.14, 95%, CI [0.93-4.90]; P=0.070), and higher among the primi– and secundigravida (6.4%) than multigravida (2.2%, HR=3.01 [1.04-8.69]; P=0.042). The PCR-adjusted failure risk was 1.1% overall (Mali 0.8%, Burkina-Faso 1.4%). The frequencies (95% CI) of the *dhfr* double *and* triple mutant and *dhps* 437 and 540 alleles mutant genotype at enrolment were 24.2% (23.7-25.0), 4.7% (4.4-5.0), and 21.4% (20.8-22.0) and 0.37% (0.29-0.44) in Mali, and 7.1% (6.5-7.7), 44.9% (43.8-46.0) and 75.3% (74.5-76.2) and 0% in Burkina-Faso, respectively. There were no *dhfr* 164L or *dhps* 581G mutations.

**Conclusion:**

SP remains effective at clearing existing infections when provided as IPTp to asymptomatic pregnant women in Mali and Burkina. Continued monitoring of IPTp-SP effectiveness, including of the impact on birth parameters in this region is essential.

## Background

In sub-Saharan Africa, malaria places 31 million pregnancies at risk of maternal anaemia and intrauterine growth retardation resulting in low birth weight (LBW) annually [1-3]. In this region, the World Health Organization (WHO) recommends Intermittent Preventive Treatment in pregnancy (IPTp) with at least two doses of sulphadoxine-pyrimethamine (SP) for the control of malaria in pregnancy [4]. The two-dose IPTp-SP regimen has been shown to be very effective and is associated with an average reduction in the risk of LBW of 29% [2]. More recent meta-analysis has shown that this can be enhanced further by providing three or more doses of SP at monthly intervals during pregnancy [5].

However, the emergence of SP resistance is potentially reducing the effectiveness of SP. In the early 2000s, SP was abandoned as first line treatment for symptomatic malaria in the general population in sub-Saharan Africa in favour of more effective artemisinin-based combination therapy (ACT). Because IPTp with SP continued to provide significant protection in areas with moderate to high parasite resistance [2], SP continues to be recommended by WHO for IPTp, and is currently the only anti-malarial used for this indication [6]. The degree of SP resistance correlates with the frequency of single nucleotide polymorphisms (SNPs) that encode amino acid substitutions in the dihydrofolate reductase (*dhfr*) and dihydropteroate synthetase (*dhps*) genes of *Plasmodium falciparum*. High grade resistance is a particular concern in eastern and southern Africa [7], where high frequencies of parasites bearing haplotypes with three mutations in *dhfr* (encoding the N51I, C59R, and S108N) and two in *dhps* (encoding the A437G and K540E substitutions) exist, especially if the additional *dhfr*164L or *dhps*581G mutations occur [8,9]. The latter has recently been associated with poor birth outcomes in IPTp-SP recipients [10], although this association has not yet been confirmed in other studies in eastern and southern Africa [11-13].

In contrast, the parasite populations in western Africa seem to be mostly sensitive to SP [7,14-16], and IPTp-SP has proven to be highly effective and efficacious in clinical trials and observational studies [5,15]. However, spread of SP resistance from eastern and southern Africa, or the *de novo* development of high-level SP resistance may occur and monitoring of the effectiveness of SP when employed as IPTp is essential.

Despite this need, there are no internationally standardized methods to evaluate the *in vivo* effectiveness of IPTp-SP. Furthermore, the relationship between the level of SP resistance as measured by molecular markers and impact of IPTp-SP on birth parameters, or the treatment response in asymptomatic women receiving SP for IPTp is not known. Hitherto, monitoring SP resistance was predominantly based on *in vivo* treatment responses among symptomatic children with acute malaria. However, extrapolation from children to asymptomatic pregnant women is not appropriate as protective immunity against *P. falciparum* malaria is acquired progressively with cumulative exposure and age. As a result pregnant women in endemic areas remain typically asymptomatic when infected and have lower parasites densities than sick children and as a results have better treatment responses to anti-malarials, including SP [17,18]. A single arm 42 days *in vivo* efficacy study of SP was conducted to determine the parasitological treatment response to SP and the duration of post-treatment prophylaxis among asymptomatic parasitaemic women receiving SP for IPTp in, Mali and Burkina Faso. The prevalence of molecular markers for SP resistance was also assessed to explore the relationship between the level of resistance and the treatment responses.

## Methods

### Study sites and study period

**Figure 1:**
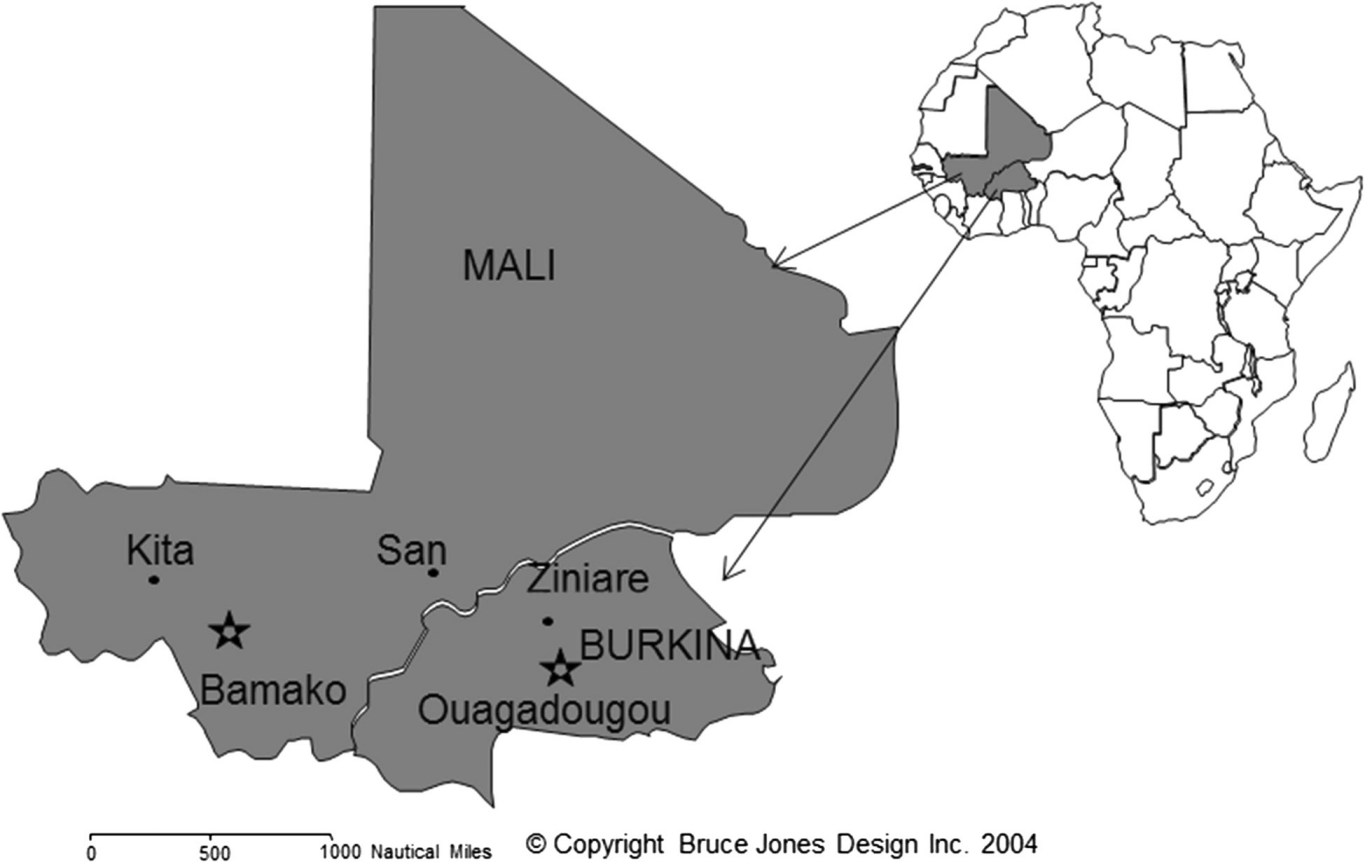
Study sites in Mali (Kita and San) and Burkina Faso (Ziniaré).

In Mali, the study was conducted from July 2009 to March 2010 in 2 district health centres located in the towns of Kita in the Kayes region in western Mali and in San in the Segou region situated approximately 500 kilometres east of Kita (Figure 1). Malaria transmission in the two sites is typical for most of the Sahel region with highly seasonal transmission restricted to a single period of three to five months during and shortly after the rainy season, with peak transmission in October. The degree of SP resistance is low in these areas and the quintuple *dhfr/dhps* haplotype has not been found yet [15], although the *dhps*581G mutation has been described in isolation of other mutations in other settings [19,20]. No previous in-vivo studies among pregnant women were conducted in Mali.

In Burkina Faso, the study was conducted from January 2010 to December 2011 in five recruitment centres in Ziniaré town, Oubritenga Province, located 400 km South-East of the town of San (one of the study sites in Mali). Malaria transmission is seasonal peaking in September-October. In 2003, the polymerase chain reaction (PCR)-adjusted parasitological failure rate by day-28 was 13% among symptomatic primi- and secundigravida with acute falciparum malaria in Ouagadougou, located 50 kilometers from the study site [16].

### Participants and procedures

In both countries, pregnant women of all parities with a gestational age between 16–30 weeks attending for antenatal care for their first dose of IPT-SP were included. Women were screened for malaria infection using HRP2 and pLDH-based combo Rapid Diagnostic Tests (RDTs, CareStart™Malaria HRP-2/pLDH[Pf/pan] Combo Test) [21,22]. Women with a positive RDT were then screened for malaria parasitaemia by microscopy and eligible for enrolment if they had a positive blood smear, were asymptomatic, were willing to participate in the six-week follow-up and provided written individual informed consent. Women were excluded if they had a history of hypersensitivity to SP or its components, a history of prior use of IPTp-SP during this pregnancy, or a history of receipt of other anti-malarials or antibiotics with anti-malarial activity in the previous month.

On enrolment, clinical, obstetric and demographic data were obtained and information on bed net type and use recorded. A finger-prick blood sample was taken for malaria smears, haemoglobin assessment, and dried blood spots (DBSs) for parasite DNA.

Three tablets of SP containing a total dose of 1,500 mg sulphadoxine and 75 mg of pyrimethamine were administered as a single dose on day 0 by the study staff. If vomiting occurred within 30 minutes after administration, the full dose was re-administered. Women were scheduled to be seen again weekly from day 7 onwards for 42 days for a brief clinical exam, assessment of the axillary temperature and collection of blood by finger prick for malaria smears, RDT, and DBSs for PCR. Participants were asked to return to the study clinic any time they felt ill in between the scheduled visits. Women with positive smear or severe malaria at any time on or after day 4 were treated according to national guidelines.

In Mali, the study drug used was manufactured by Kinapharma limited Ltd, Ghana and in Burkina Faso this was also from Kinapharma limited Ltd, Ghana and Medreich limited, India. A sample of 50 tablets from each batch was assessed for quality using high-performance liquid chromatography (HPLC) conducted in Atlanta, GA, USA by the US Centers for Disease Control and Prevention (CDC) to determine the amount of the active ingredient and the dissolution profile. Both brands passed the dissolution and content analyses criteria set by the United States Pharmacopeia (USP).

### Laboratory methods

Haemoglobin concentrations were measured using HemoCue® (301 System) on days 0, 14, 28 and 42, and on the day of parasite recurrence. Giemsa-stained blood smears were assessed in duplicate and if a discrepancy was found (positive *vs* negative) the smear was read by a third expert microscopist. Asexual parasites were counted against 300 leukocytes and densities expressed per mm^3^ of blood assuming a leucocyte count of 7,500/mm^3^. Smears were declared negative if no parasites were detected in 100 high-power fields.

PCR assays were performed in the laboratories of the Gillings School of Global Public Health, University of North Carolina, Chapel Hill, NC, USA using genomic DNA (gDNA) extracted from dried blood spots (DBSs) stored on Whatman 3 MM filter papers to differentiate between recrudescence and new infection in follow-up specimens with parasite recurrence. A standard method was employed to genotype parasites using polymorphisms of the merozoite surface protein-1 (*msp-1*), merozoite surface protein-2 (*msp-2*), and glutamate rich protein (*glurp*) genes [23]. The prevalence of genomic markers of parasite SP resistance, genomic DNA from all parasitaemic women was pooled by study site (2 in Mali and 1 in Burkina Faso). Fragments of the *dhfr* and *dhps* genes containing the SNPs of interest were PCR-amplified from the pooled gDNA from each site to produce a mixture of gene fragments [24], and these PCR products were sequenced on a Roche GS Junior next-generation sequencing system.

### Study endpoints classification

The primary outcome was the PCR-unadjusted % of patients with parasites recurrence by day 42, defined as a positive diagnostic test (by microscopy) for malaria at any visit between days 4 and 42. To define treatment failure, the standard WHO criteria [25] were used.

### Statistical analysis

Data were analysed using STATA v12 and SPSS version 20. The treatment responses are summarized by weeks of follow-up. The therapeutic response was estimated using the Kaplan-Meier product limit formula [26]. In the PCR-unadjusted analysis, recurrences were treated as treatment failures and all other events (e.g. withdrawal or protocol deviations) resulted in censoring at the time of that event, or at the time of their last follow-up visit in case of loss to follow-up. A similar strategy was used for the PCR-adjusted analysis except that patients with new *P. falciparum* infections (reinfections) were censored at the time of parasite reappearance [26].

### Ethical considerations

The protocol was approved by the Faculty of Medicine, Pharmacy and Dentistry, University of Bamako, Mali, Institutional Ethical Review Committee, the National Ethical Review Committee and Ministry of Health, Burkina-Faso, the University of North Carolina, USA, and the Liverpool School of Tropical Medicine, UK.

## Results

### Treatment responses

**Figure 2:**
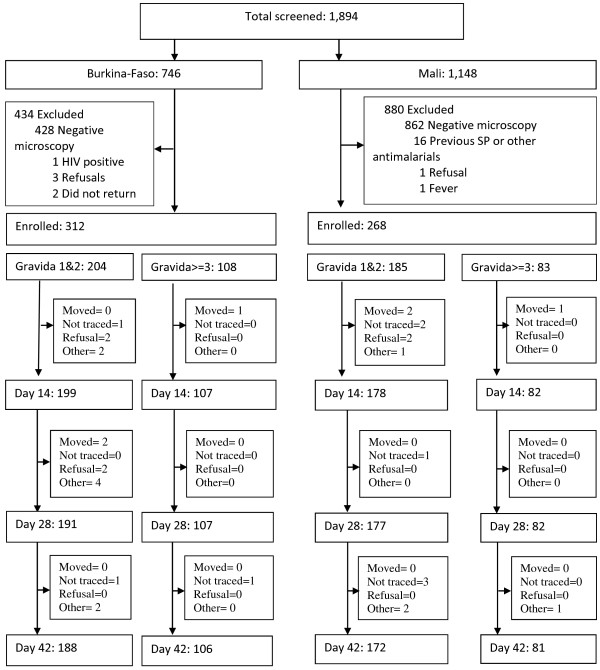
Study flow chart.

Overall 580 of 584 women who fulfilled all enrollment criteria were enrolled (99.3%, Figure 2 and Table 1), and 572 of the 580 contributed to the survival analysis. Eight of the 33 women lost to follow-up were not seen after day 0; 3 from Mali and 5 from Burkina-Faso. The remaining 25 were censored on the day they were last seen.

**Table 1:** **Baseline characteristics of women enrolled in SP *in vivo *** **efficacy study, Burkina-Faso and Mali**

	**Burkina-Faso**	**Mali**	**All**
	**N=312**	**N =268**	**N=580**
Age, years,			
Mean (SD)	23.6 (5.4)	21.1 (5.1)	22.5 (5.4)
Residing in rural area, n (%)	81 (30.2)	146 (45.2)	222 (38.3)
Knows the date of LMP, n (%)	44 (16.4)	61 (19.6)	105 (18.1)
Pregnancy number			
Median (range)	2 (1–8)	2 (1–9)	2 (1–9)
First or second pregnancy, n (%)	204 (65.4)	185 (69.0)	389 (67.1)
Use of a bed net last night^a^			
Any net, n (%)	187 (60.1)	207 (77.2)	394 (68.1)
ITN, n (%)	171 (55.0)	180 (67.2)	351 (60.6)
Use medicine in first trimester			
Any medicine, n (%)	6 (1.9)	25 (9.3)	31(5.3)
Antimalarial, n (%)	2 (0.6)	15 (5.6)	17 (2.9)
Fundal height, cm			
Mean (SD)	21.5 (2.9)	21.8 (3.2)	21.7 (3.1)
Gestational age, weeks			
Mean (SD)	25.3 (3.1)	25.4 (3.2)	25.3 (3.1)
Maternal height, cm			
Mean (SD)	162.7 (6.2)	162.2 (6.3)	162.4 (6.2)
Maternal weight, kgs			
Mean (SD)	57.8 (7.5)	56.4 (8.5)	57.2 (8.0)
Haemoglobin, g/dL^b^			
Mean (SD)	10.1 (1.4)	9.6 (1.6)	9.9 (1.5)
Anaemia (Hb <11 /dL), n (%)	225 (72.4)	198 (80.5)	423 (75.9)
Moderate-Severe anaemia (Hb <8g/dL),	22 (7.1)	40 (16.3)	62 (11.1)
n (%)			
Peripheral parasitaemia	623	716	664
GMPD/μl (95% CI)	(537–723)	(598–859)	(592–746)

Notes: Data are n0. (%), unless otherwise indicated.

N, sample size; n, number of events; SD, Standard deviation; LMP, Last Menstrual Period; ITN, Insecticide Treated Net; cm, centimeters; kgs, kilograms; g/dL, Gram per deci-Litre; Hb, Haemoglobin; GMPD/μl, Geometric Mean Parasite Density per microlitre.

^a^Bed net use was not evaluated in 1 subject from Burkina-Faso.

^b^Haemoglobin was not measured for 1 subject in Burkina-Faso and 22 subjects in Mali.

**Figure 3:**
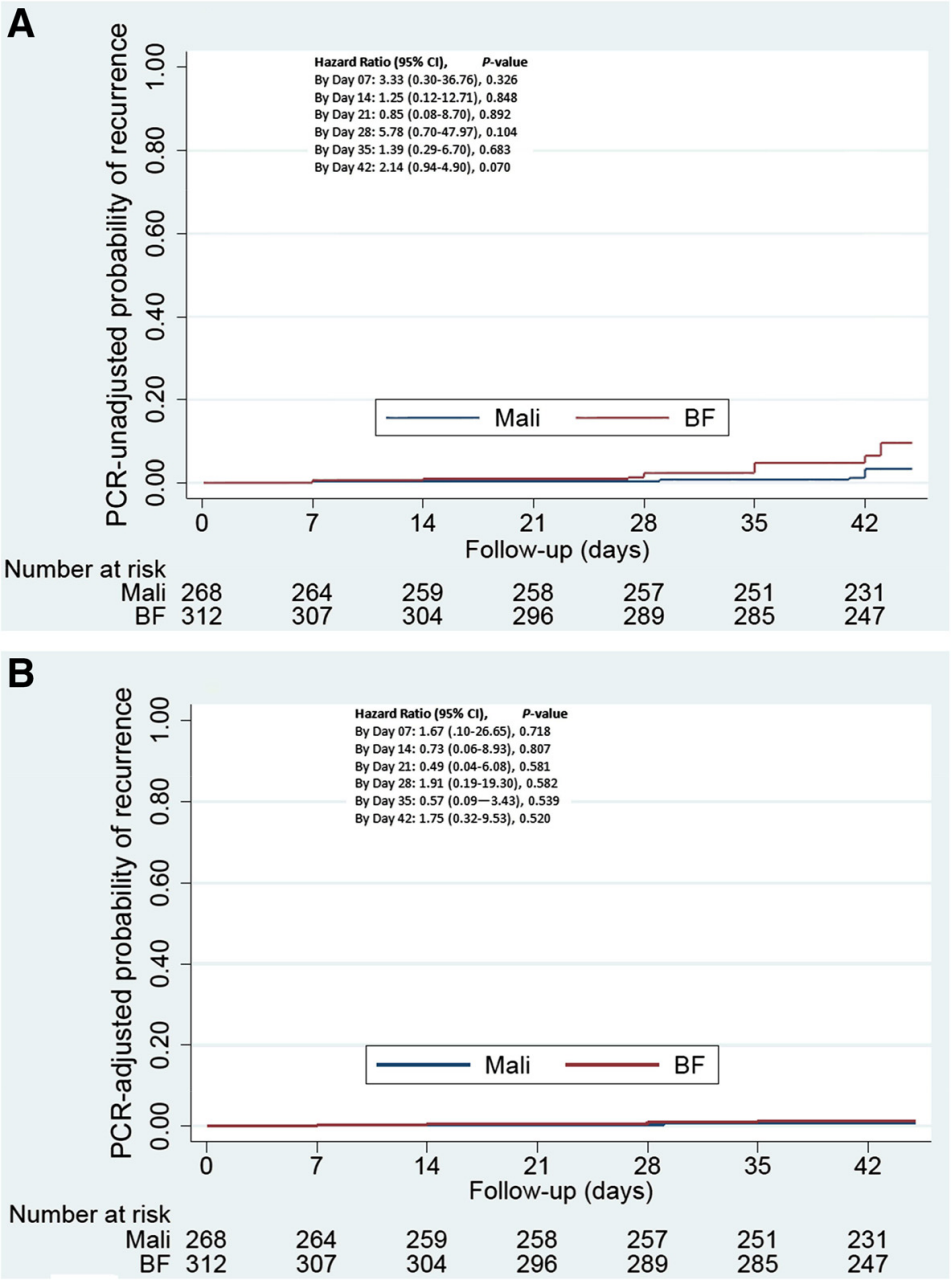
**Probability of parasitological failure by microscopy in Burkina-Faso and Mali.** Notes: This graph shows the crude and PCR adjusted risk of parasitological failure in Mali and Burkina-Faso. Treatment failure was defined according to the standard WHO criteria and the cumulative risk of recurrence was determined using Kaplan-Meier survival analysis. Blue lines represent Mali and red lines Burkina-Faso. Panel **A** and panel **B** represent survival analysis for crude and PCR adjusted analysis, respectively.

**Figure 4:**
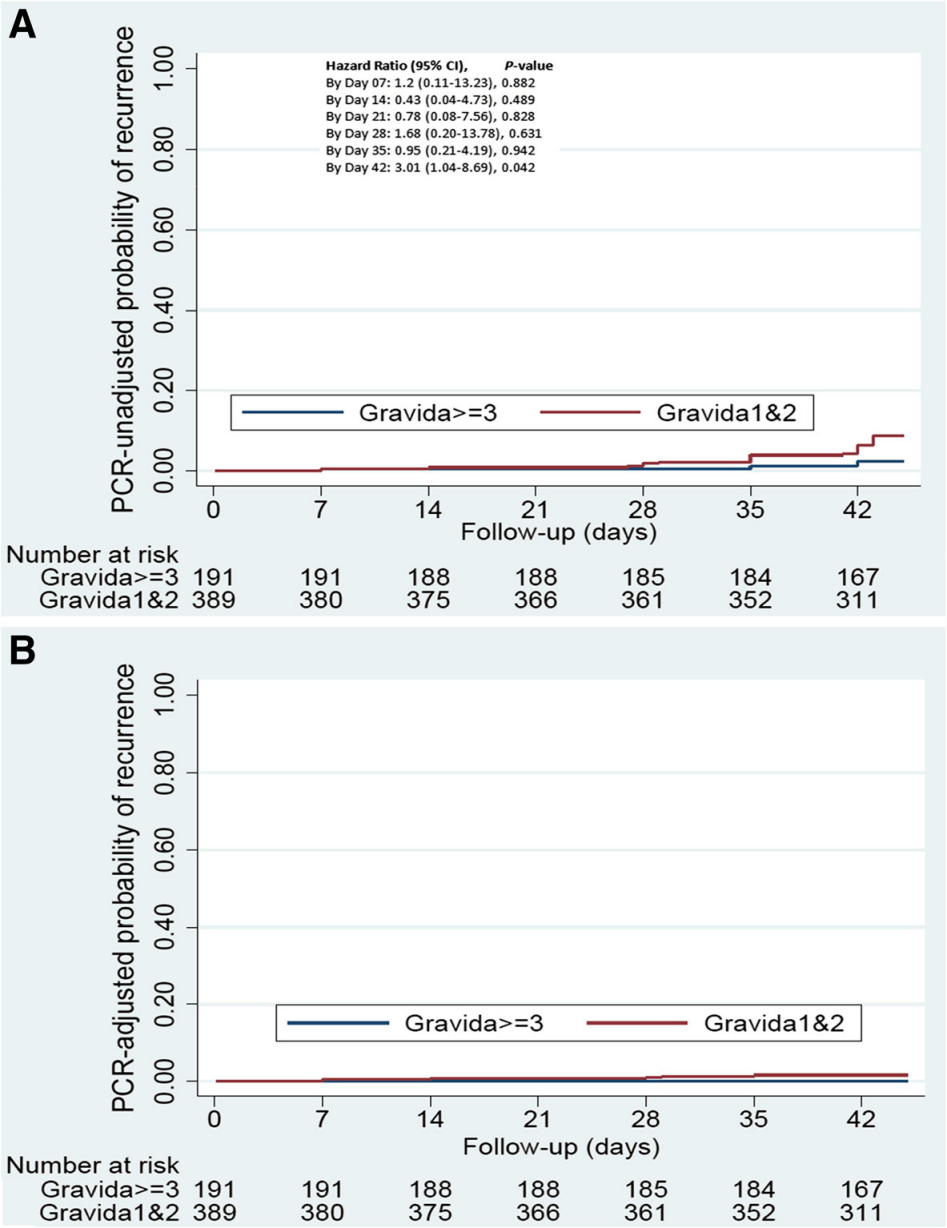
**Probability of parasitological failure by microscopy by gravida group.** Notes: (Gravidae 1&2, primi-secundigravida; Gravidae>=3, multigravida): PCR unadjusted **(Panel A)** and PCR adjusted **(Panel B)**. This graph shows the crude and PCR adjusted risk of parasitological failure in both primi-secundigravida and multigravida using Kaplan-Meier survival analysis. Blue lines represents multigravida (gravidae>=3) and the red lines represent primi-secundigravida (gravidae 1&2), respectively.

*PCR-unadjusted efficacy:* Based on microscopy, overall 27 of the 572 women had a recurrence of parasitaemia by the end of follow-up (Mali 8; Burkina Faso 19). The cumulative recurrence risks by day 42 estimated by survival analysis were 4.9% overall, and 3.2% and 6.5% in Mali and Burkina Faso respectively (Hazard Ratio [HR] Burkina vs Mali=2.14, 95% CI 0.93-4.90; P=0.070, Table 2 and Figure 3). The recurrence risk was higher among primi- and secundigravidae (6.4%) than multi-gravidae (2.2%), HR=3.01 (1.04-8.69; P=0.042) (Figure 4).

**Table 2:** Parasitological efficacy of SP among women enrolled in Burkina-Faso and Mali

**Characteristics**	**Burkina-Faso**		**Mali**		**All**	
	**N=312**		**N =268**		**N =580**	
**PCR Days**	**Non- adjusted**	**Adjusted**	**Non- adjusted**	**Adjusted**	**Non- adjusted**	**Adjusted**
Day 7:						
Number at risk	307	307	265	265	572	572
Failures, n (%)						
ETF	0	0	0	0	0	0
LCF	0	0	0	0	0	0
LPF	2 (0.5)	1 (0.3)	1 (0.4)	1 (0.4)	3 (0.5)	2 (0.3)
ACPR n (%)	305 (99.5)	306 (99.7	264 (99.6)	264 (99.6)	569 (99.5)	570 (99.7)
Day 14:						
Number at risk	306	306	260	260	566	566
Failures n (%)						
ETF	0	0	0	0	0	0
LCF	0	0	0	0	0	0
LPF	3 (1.0)	2 (0.7)	1 (0.4)	1 (0.4)	4 (0.7)	3 (0.5)
ACPR n (%)	303 (99.0)	304 (99.3	259 (99.6)	259 (99.6	562 (99.3)	563 (99.5)
Day 21:						
Number at risk	300	300	259	259	559	559
Failures n (%)						
ETF	0	0	0	0	0	0
LCF	0	0	0	0	0	0
LPF	3 (1.0)	2 (0.7)	1 (0.4)	1 (0.4)	4 (0.7)	3 (0.5)
ACPR n (%)	297 (99.0)	298 (99.3)	258 (99.6)	258 (99.6)	542 (99.5)	556 (99.5)
Day 28:						
Number at risk	297	297	259	259	556	556
Failures n (%)						
ETF	0	0	0	0	0	0
LCF	0	0	0	0	0	0
LPF	7 (2.4)	3 (1.0)	2 (0.8)	2 (0.8)	9 (1.6)	5 (0.9)
ACPR n (%)	290 (97.6)	294 (99.0)	257 (99.2)	257 (99.2)	547 (98.4)	551 (99.1)
Day 35:						
Number at risk	295	294^a^	253	254	548	547^a^
Failures n (%)						
ETF	0	0	0	0	0	0
LCF	0	0	0	0	0	
LPF	14 (4.8)	4 (1.4)	2 (0.8)	2 (0.8)	16 (2.9)	6 (1.1)
ACPR n (%)	281 (95.2)	290 (98.6)	251 (99.2)	252 (99.2)	532 (97.1)	541 (98.9)
Day 42:						
Number at risk	293	292^a^	253	251^a,b^	546	544^c^
Failures n (%)						
ETF	0	0	0	0	0	0
LCF	0	0	0	0	0	0
LPF	19 (6.5)	4 (1.4)	8 (3.2)	2 (0.8)	27 (4.9)	6 (1.1)
ACPR n (%)	274 (93.5)	288 (98.6)	245 (96.8)	249 (99.2)	519 (95.1)	538 (98.9)
Median (range) time in days	35 (7–43)^d^	21 (7–35)^e^	42 (7–42)^d^	18 (7–29)^e^	35 (7–43)^d^	21 (7–35)^e^

Notes: PCR, Polymerase Chain Reaction; ETF, Early Treatment Failure; LCF, Late Treatment Failure; LPF, Late parasitological Failure; ACPR, Adequate Clinical and Parasitological Response.

^a^One PCR inconclusive.

^b^PCR analysis not conducted for 1 woman with recurrent parasitaemia in Mali.

^c^PCR inconclusive (1 from Mali, 1 from Burkina) and no PCR analysis available (1 from Mali). These three cases were censored in the survival analysis.

^d^Median (range) time to reinfection.

^e^Median (range) time to PCR-adjusted failure.

*PCR-adjusted efficacy:* From 26 of the 27 recurrences, DNA could be extracted and 24 were genotyped successfully. This suggested that only 6 of the 24 were recrudescences. The PCR-adjusted cumulative failure rate obtained by survival analysis was 1.1% overall, and 0.8% in Mali and 1.4% in Burkina-Faso (Figure 3). Overall, median (range) time to PCR-adjusted failure and to reinfection was 21 (7–35) and 35 (7–43) days, respectively.

**Figure 5:**
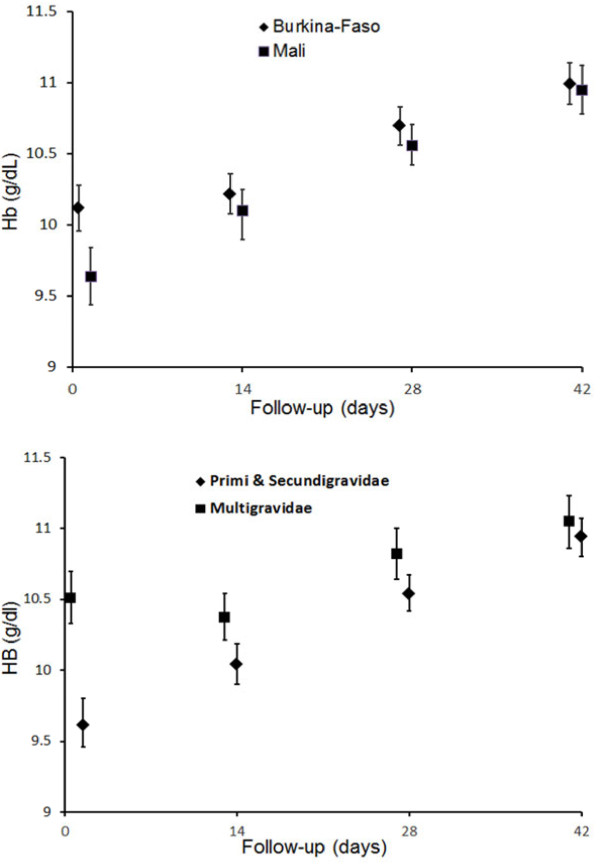
**Increase in haemoglobin concentrations by country in all gravida.** Notes: (top panel) and by gravidae group (bottom panel). Analysis was done with repeated measures Generalized Estimating Equation (GEE), adjusted for the baseline hemoglobin levels on Day-0. Black squares or diamonds represent the point estimates and vertical lines the corresponding 95% confidence intervals.

*Haematological response:* There was a significant increase in the mean haemoglobin concentrations compared to enrolment at all-time points measured in both countries and both among primi-, secundi- and multigravida (Figure 5 and Table 3).

**Table 3:** Haemoglobin concentration and anaemia among women enrolled in Burkina-Faso and Mali

**Characteristics**	**Burkina-Faso**	**Mali**	**All**
Day 0			
N	311	246	557
Mean haemoglobin (SD), g/dl	10.1 (1.4)	9.6 (1.6)	9.9 (1.5)
Anaemia (<11 g/dl), n (%)	225 (72.4)	198 (80.5)	423 (75.9)
Day 14			
N	301	237	538
Mean haemoglobin (SD), g/dl	10.2 (1.3)	10.1 (1.4)	10.2 (1.3)
Anaemia (<11 g/dl), n (%)	208 (69.1)	177 (74.7)	385 (71.6)
Mean difference, 95% CI^a^	0.13 (0.004, 0.26)	0.44 (0.28, 0.59)	0.26 (0.16, 0.36)
Risk ratio, 95% CI^b^	0.96 (0.86, 1.06)	0.93 (0.84, 1.02)	0.94 (0.88, 1.01)
Day 28			
N	290	244	534
Mean haemoglobin (SD), g/dl	10.7 (1.2)	10.6 (1.2)	10.6 (1.2)
Anaemia (<11 g/dl), n (%)	171 (59.0)	153 (62.7)	325 (60.7)
Mean difference, 95% CI^a^	0.60 (0.46,0.74)	0.87 (0.69, 1.06)	0.72 (0.61, 0.83)
Risk ratio, 95% CI^b^	0.82 (0.72, 0.92)	0.78 (0.70, 0.87)	0.80 (0.74, 0.87
Day 42			
N	265	249	514
Mean haemoglobin (SD), g/dl	11.0 (1.2)	10.9 (1.3)	10.9 (1.3)
Anaemia (<11g/dl), n (%)	127 (47.9)	120 (48.2)	247 (48.1)
Mean difference, 95% CI^a^	0.87 (0.65, 1.09)	1.30 (1.11, 1.49)	1.06 (0.93, 1.18)
Risk ratio, 95% CI^b^	0.66 (0.57, 0.77)	0.60 (0.52, 0.69)	0.63 (0.57, 0.70)

Notes:

N, sample size; n, number of events; SD, standard deviation; g/dl, gram per decilitre; CI, confidence interval.

^a^Mean difference and 95% confidence interval for each time that haemoglobin was measured using day 0 as reference category.

^b^Risk ratio and 95% confidence interval for each time that haemoglobin was measured using day 0 as reference category, adjusted for gravida and site (all), and for gravida (in each country).

### Prevalence of molecular markers for SP resistance at booking

**Figure 6:**
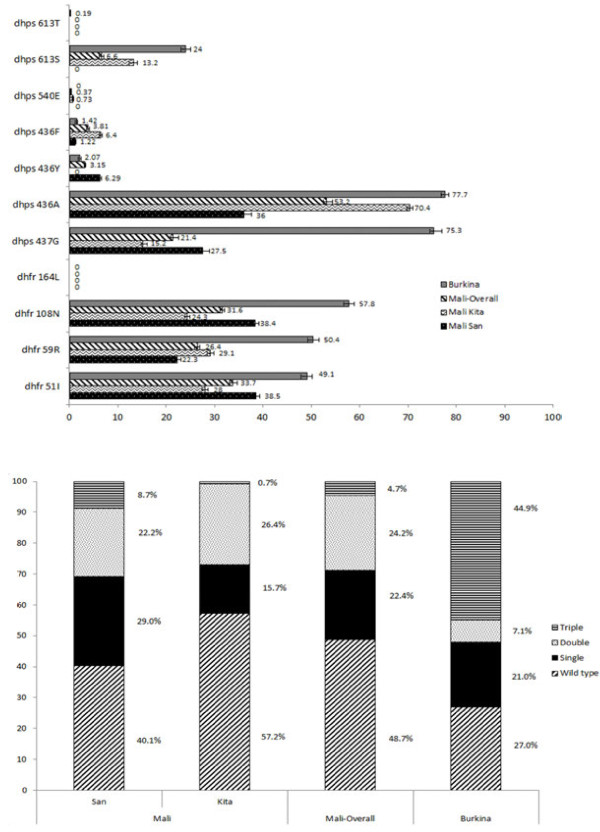
**Prevalence of SP resistance molecular makers in Burkina-Faso and Mali among parasitaemic women at their antenatal booking visit (pre-SP).** Notes: *dhfr* /*dhps* alleles (Top panel) and *dhfr* haplotypes (Bottom panel). Mutant allele frequencies are represented in the top panel graph by horizontal bars. Lines depict the 95% confidence intervals. The presence of “0” represents the absence of point mutations for a designed codon. The bottom panel represents the frequency of *dhfr* haplotypes (N51I, C59R, and S108N) per country.

No *dhfr* 164L or *dhps*581G mutations were found in any of the three sites; the *dhps* 540E mutation was found in one site of the two sites Mali, but at a very low prevalence (Figure 6).

## Discussion

SP when given as IPTp to asymptomatic parasitaemic pregnant women was associated with a high cure rate and marked increases in haemoglobin concentrations by day 42 in the 3 study sites in Mali and Burkina-Faso. Overall, only 4.9% of women had a recurrence of parasites by day 42, and genotyping suggested that the vast majority of these were reinfections. Overall only 1.1% of treatments resulted in true treatment failures (recrudescence) and all of these were asymptomatic. The study shows that SP remains very effective at clearing existing infections when used as IPTp for malaria prevention in Mali and Burkina-Faso. This study also showed the potential value of using in-vivo follow-up to assess the parasitological cure rates among parasitaemia asymptomatic pregnant women who are due for their first dose of SP for IPTp.

The pooled molecular assays for the surveillance of SP resistance showed that almost 50% of the parasite population in Burkina Faso, but only 9% in San and <1% in Kita, carried the *dhfr* triple mutations. The pooled deep sequencing of *P. falciparum* parasitaemia can provide estimates of the mutant allele frequencies, but does not provide estimates of the quadruple and quintuple *dhfr/dhps* haplotypes. Nevertheless, the *dhps* 540E mutation, which is a proxy for the quintuple haplotype conferring mid-level resistance to SP, was present in only one of the two sites of Mali and at very low frequency (0.73%, 95% CI 0.58-0.87). The mutation at *dhfr* codon 164L and *dhps* codon 581G conferring very high-level resistance to SP were absent. In addition, there were several novel mutations in *dhps,* which were limited to a very low frequency. Their clinical and biological significance is unknown, but their quantification underscores the ability of the pooled genotyping approach to uncover low-level subpopulations of parasites.

The 1.4% failure rate in Burkina Faso among asymptomatic women in this study is in contrast to the 13% PCR-adjusted failure rate by day-28 observed in the previous in-vivo study among symptomatic pregnant women conducted in 2003 in an area located ≈32 miles south from the current site [16]. The average parasite densities in the previous study were 10 fold higher than in the current study, illustrating the differences in treatment responses when SP is used as IPTp in asymptomatic women with predominantly low-grade parasitaemia *vs.* acutely ill women requiring case-management drugs. This may in part explain the earlier findings from randomized controlled trials that IPTp-SP remained surprisingly effective in areas with moderate to high levels of SP resistance [2,5].

The study provides an important contribution to the understanding of the predictive value of the frequency of population estimates of the different *dhfr* and *dhps* mutations on the efficacy of SP in clearing malaria infection among asymptomatic pregnant women, especially when our results are compared against day 42 failure rates in areas with higher resistance. For example, the *dhfr* triple mutation (Ile51+Arg59+Asn108) was present in almost 50% of the parasite population in Burkina Faso yet only 1.4% of the treatments recrudesced by day 42. The *dhfr* triple mutation is known to confer intense pyrimethamine resistance in vitro [27] and is associated with an approximate 1,000-fold reduction in pyrimethamine susceptibility [28] and with an increased risk of SP treatment failure in children with acute malaria [29-31]. These combined data suggests that parasite densities and immunity contribute importantly to parasite clearance, which in turn influences the association of treatment outcome with *dhfr* and *dhps* alleles.

It is likely that the results of this study are representative for large parts of West and Central Africa that have a similar low geographic prevalence of the *dhfr/dhps* quadruple or quintuple mutations reflecting low and mid-level resistance to SP [7]. A key question is whether this situation can be sustained or whether further development of SP drug resistance is inevitable in this region. Mutations arise under antifolate pressure in a stepwise fashion, with successive mutations conferring higher levels of resistance [32]. Previous studies from Ghana showed a rapid increase in the prevalence of the triple-mutant *dhfr* alleles among *falciparum* isolated from pregnant women in an area where pyrimethamine prophylaxis (as mono-therapy) was used 6 to 8 years previously for the prevention of malaria [33]. Some fitness-reducing mutations, such as the *dhfr* I164L can only be sustained under conditions of sustained drug pressure. The switch from SP as first-line treatment for symptomatic malaria in the general population to an ACT will have hada marked impact on reducing SP drug pressure in the population [34]. However, the effect of continued use of cotrimoxazole in the treatment of diarrheal and respiratory infectious diseases in children should also be considered, although this drug did not appear to select for SP-resistance parasites [19]. Modelling of the impact of the introduction of IPTi in infants suggest that use of SP in small target populations such as infants or pregnant women may not sustain sufficient drug pressure to impact on the spread of drug resistance. This was also suggested in field studies in Mali [35]. However, many West African countries including Mali and Burkina are seeking to implement Seasonal Malaria Chemoprevention (SMC) [36] in children which would provide presumptive treatment over the course of the transmission season to a much larger fraction of the population. Although, the combination of amodiaquine (AQ) and SP is one of the main candidate anti-malarials for SMC, it is unclear if the introduction of this strategy will indeed increase SP drug pressure. The effect of SMC on drug pressure may be minimal if implemented on a large enough scale to impact on malaria transmission and the total parasite biomass in the SMC population, especially if an ACT is used as case-management for clinical episodes caused by any SP resistant parasites that may have escaped the drug action of SMC. It will be clearly important to monitor the prevalence of molecular markers of parasite resistance to SP, especially in areas where SP is used for both IPTp and SMC.

This investigation found a high prevalence of anaemia and showed that SP treatment was associated with a marked increase in mean haemoglobin levels by day 42. The fact that the impact was most pronounced in the primi,- and secundigravidae, the group most susceptible to adverse effect of malaria, may indicate that even these asymptomatic infections are an important cause of maternal anaemia in this subgroup. These findings are consistent with previous findings that showed IPTp has a marked beneficial impact on moderate-to-severe anaemia in Mali [15,37].

The study was limited by the lack of genotyping of parasites from individual women for molecular markers of SP resistance, and the genomic DNA from pooled sequencing by study site was not able to explore the correlation between treatment efficacy or the haematological response in individual women and SP resistance molecular markers.

## Conclusion

This is among the first studies to examine the 42-day *in vivo* response of IPTp-SP in asymptomatic women in areas with low level of SP resistance in West Africa. Despite growing concerns about the impact of SP resistance in east and southern Africa, this study shows that SP remains effective at clearing existing infections and improving haemoglobin concentration when provided as IPTp to asymptomatic pregnant women in Mali and Burkina-Faso. SP has many attributes that makes it an excellent candidate for IPTp, and it is thus likely that it could remain the drug of choice for IPTp in this region for the foreseeable future. However continued monitoring of SP resistance over the next years in this region coupled with monitoring of IPTp-SP effectiveness on birth parameters is essential.

## Competing interests

The authors declare that they have no competing interests.
